# Supplementary material for: Investigating the Relationship between Parental Education, Asthma and Rhinitis in Children Using Path Analysis
Source: Int J Environ Res Public Health. 2022 Nov 6;19(21):14551. doi: 10.3390/ijerph192114551 (PMC9654957; doi:10.3390/ijerph192114551)
Supplement: Supplementary file 1 [file ijerph-19-14551-s001.zip › ijerph-1982431-supplementary.pdf]

Table S1. Direct and indirect effects on current asthma defined as physician diagnosed asthma (PDA) plus wheezing or whistling in the last 12 months or PDA plus asthma medications in the last 12 months. ETS: Environmental Tobacco Smoke.

	Coefficient	*P-value*
Direct effects on current asthma (CA):		
Parental education (PE)	-0.1403	0.505
Obesity	0.3128	0.210
Crowding index	0.2992	0.185
Number of children	0.2149	0.330
Traffic	0.0089	0.972
Mould/dampness	-0.0923	0.765
Pets	-0.2280	0.365
Maternal smoking during pregnancy	0.2440	0.425
Paternal smoking during pregnancy	-0.2920	0.232
Early ETS	0.5041	0.051
Current smoker(s)	0.0593	0.813
Indirect effect of PE on CA through:		
Obesity	0.0378	0.444
Crowding index	0.3370	0.163
Number of children	0.1339	0.328
Traffic	0.0001	0.997
Mould/dampness	-0.0008	0.983
Pets	0.0039	0.868
Maternal smoking during pregnancy	0.1146	0.390
Paternal smoking during pregnancy	-0.1418	0.228
Early ETS	0.2972	0.095
Current smoker(s)	0.0415	0.808

Table S2. Direct and indirect effects on outcomes (physician diagnosed asthma, current asthma and current allergic rhinitis) after multiple imputation for missing values. ETS: Environmental Tobacco Smoke.

**Outcome:**	**Physician diagnosed asthma**		**Current asthma**		**Current allergic rhinitis**	
	Coefficient	*P-value*	*Coefficient*	*P-value*	*Coefficient*	*P-value*
Direct effects on outcome:						
Parental education (PE)	-0.0788	0.586	-0.4971	0.073	-0.0105	0.916
Obesity	0.2934	0.102	-0.0118	0.974	0.0839	0.525
Crowding index	-0.0072	0.964	-0.2859	0.358	0.0990	0.368
Number of children	0.2416	0.120	0.3823	0.182	-0.0888	0.421
Traffic	-0.0299	0.863	-0.3010	0.391	-0.0052	0.965
Mould/dampness	0.1002	0.619	0.2249	0.525	0.3707	0.005
Pets	-0.2004	0.240	0.0703	0.810	-0.2112	0.068
Maternal smoking during pregnancy	0.5345	0.010	0.4883	0.216	0.1317	0.417
Paternal smoking during pregnancy	-0.1764	0.284	-0.0015	0.996	-0.0535	0.633
Early ETS	0.3295	0.062	0.2794	0.400	0.2804	0.023
Current smoker(s)	0.0243	0.888	-0.2737	0.401	-0.1781	0.135
Direct effects of PE on:						
Obesity	0.1338	0.213	0.1338	0.213	0.1338	0.213
Crowding index	1.1052	0.000	1.1052	0.000	1.1052	0.000
Number of children	0.6247	0.000	0.6247	0.000	0.6247	0.000
Traffic	0.0166	0.866	0.0166	0.866	0.0166	0.866
Mould/dampness	0.0327	0.778	0.0327	0.778	0.0327	0.778
Pets	-0.0516	0.572	-0.0516	0.572	-0.0516	0.572
Maternal smoking during pregnancy	0.4388	0.001	0.4388	0.001	0.4388	0.001
Paternal smoking during pregnancy	0.4757	0.000	0.4757	0.000	0.4757	0.000
Early ETS	0.5743	0.000	0.5743	0.000	0.5743	0.000
Current smoker(s)	0.6972	0.000	0.6972	0.000	0.6972	0.000
Indirect effects of PE on outcome through:						
Obesity	0.0393	0.349	-0.0016	0.985	0.0112	0.719
Crowding index	-0.0079	0.956	-0.3160	0.288	0.1095	0.374
Number of children	0.1509	0.185	0.2388	0.145	-0.0555	0.383
Traffic	-0.0005	0.982	-0.0050	0.917	-0.0001	0.993
Mould/dampness	0.0033	0.891	0.0074	0.893	0.0121	0.789
Pets	0.0103	0.608	-0.0036	0.913	0.0109	0.546
Maternal smoking during pregnancy	0.2345	0.032	0.2143	0.186	0.0578	0.479
Paternal smoking during pregnancy	-0.0839	0.356	-0.0007	0.997	-0.0254	0.621
Early ETS	0.1892	0.067	0.1605	0.420	0.1610	0.030
Current smoker(s)	0.0170	0.896	-0.1909	0.400	-0.1242	0.152

Table S3. Direct and indirect effects on outcomes (physician diagnosed asthma, current asthma and current allergic rhinitis) using quantitative variable for crowding index. ETS: Environmental Tobacco Smoke.

**Outcome:**	**Physician diagnosed asthma**		**Current asthma**		**Current allergic rhinitis**	
	Coefficient	*P-value*	*Coefficient*	*P-value*	*Coefficient*	*P-value*
Direct effects on outcome:						
Parental education (PE)	0.0586	0.721	-0.3651	0.258	-0.0335	0.770
Obesity	0.2501	0.213	0.1451	0.711	0.1214	0.409
Crowding index	-0.0969	0.605	-0.0493	0.891	0.1456	0.207
Number of children	0.3181	0.065	0.3690	0.261	-0.0657	0.595
Traffic	0.0882	0.647	-0.0149	0.969	0.0743	0.584
Mould/dampness	0.0696	0.764	-0.0168	0.970	0.3991	0.009
Pets	-0.3047	0.124	0.0815	0.815	-0.2354	0.079
Maternal smoking during pregnancy	0.5055	0.032	0.7140	0.105	0.0473	0.801
Paternal smoking during pregnancy	-0.1052	0.578	-0.0092	0.980	-0.0605	0.645
Early ETS	0.3602	0.078	0.3263	0.412	0.3410	0.019
Current smoker(s)	-0.0547	0.783	-0.5080	0.194	-0.1673	0.228
Direct effects of PE on:						
Obesity	0.1207	0.277	0.1207	0.277	0.1207	0.277
Crowding index	0.2465	0.000	0.2465	0.000	0.2465	0.000
Number of children	0.6229	0.000	0.6229	0.000	0.6229	0.000
Traffic	0.0154	0.878	0.0154	0.878	0.0154	0.878
Mould/dampness	0.0085	0.942	0.0085	0.942	0.0085	0.942
Pets	-0.0172	0.855	-0.0172	0.855	-0.0172	0.855
Maternal smoking during pregnancy	0.4697	0.000	0.4697	0.000	0.4697	0.000
Paternal smoking during pregnancy	0.4854	0.000	0.4854	0.000	0.4854	0.000
Early ETS	0.5897	0.000	0.5897	0.000	0.5897	0.000
Current smoker(s)	0.6998	0.000	0.6998	0.000	0.6998	0.000
Indirect effects of PE on outcome through:						
Obesity	0.0302	0.470	0.0175	0.796	0.0146	0.588
Crowding index	-0.0239	0.622	-0.0122	0.919	0.0359	0.191
Number of children	0.1982	0.114	0.2298	0.244	-0.0409	0.574
Traffic	0.0014	0.960	-0.0002	0.995	0.0011	0.945
Mould/dampness	0.0006	0.975	-0.0001	0.999	0.0034	0.937
Pets	0.0053	0.832	-0.0014	0.949	0.0041	0.831
Maternal smoking during pregnancy	0.2374	0.021	0.3354	0.103	0.0222	0.764
Paternal smoking during pregnancy	-0.0511	0.600	-0.0045	0.980	-0.0294	0.554
Early ETS	0.2124	0.079	0.1924	0.470	0.2011	0.026
Current smoker(s)	-0.0383	0.772	-0.3555	0.228	-0.1171	0.192

Table S4. Direct and indirect effects on outcomes (physician diagnosed asthma, current asthma and current allergic rhinitis) changing the cut-off for number of children in the family to 'one child' vs 'two or more children' or using actual quantitative variable. ETS: Environmental Tobacco Smoke.

**Number of children:**	**One child vs 2 or more**						**Actual number**					
**Outcome:**	**Physician diagnosed asthma**		**Current asthma**		**Current allergic rhinitis**		**Physician diagnosed asthma**		**Current asthma**		**Current allergic rhinitis**	
	Coefficient	*P-value*	*Coefficient*	*P-value*	*Coefficient*	*P-value*	*Coefficient*	*P-value*	*Coefficient*	*P-value*	*Coefficient*	*P-value*
Direct effects on outcome:												
Parental education (PE)	0.0668	0.685	-0.2786	0.387	-0.0317	0.782	0.0352	0.831	-0.3267	0.312	-0.0303	0.791
Obesity	0.2620	0.192	0.1365	0.727	0.1195	0.417	0.2628	0.191	0.1440	0.714	0.1268	0.389
Crowding index	0.0299	0.864	-0.2128	0.538	0.1084	0.381	-0.0199	0.913	-0.2979	0.409	0.0742	0.559
Number of children	0.5205	0.079	0.7334	0.231	-0.1135	0.511	0.1947	0.030	0.2681	0.101	0.0300	0.656
Traffic	0.0780	0.685	-0.0413	0.914	0.0768	0.571	0.0929	0.630	-0.0264	0.945	0.0785	0.563
Mould/dampness	0.0716	0.758	0.0084	0.985	0.4006	0.009	0.0675	0.771	0.0091	0.984	0.4007	0.009
Pets	-0.2870	0.147	0.0727	0.835	-0.2428	0.070	-0.3054	0.123	0.0481	0.890	-0.2430	0.070
Maternal smoking during pregnancy	0.5125	0.030	0.7306	0.097	0.0465	0.804	0.4998	0.034	0.7193	0.102	0.0507	0.787
Paternal smoking during pregnancy	-0.1240	0.513	-0.0402	0.912	-0.0554	0.673	-0.1026	0.589	-0.0108	0.976	-0.0543	0.679
Early ETS	0.3820	0.061	0.3526	0.375	0.3360	0.021	0.3631	0.076	0.3305	0.407	0.3346	0.022
Current smoker(s)	-0.0553	0.781	-0.4674	0.231	-0.1645	0.236	-0.0663	0.739	-0.4926	0.208	-0.1684	0.225
Direct effects of PE on:												
Obesity	0.1207	0.277	0.1207	0.277	0.1207	0.277	0.1207	0.277	0.1207	0.277	0.1207	0.277
Crowding index	1.1263	0.000	1.1263	0.000	1.1263	0.000	1.1263	0.000	1.1263	0.000	1.1263	0.000
Number of children	0.1249	0.324	0.1249	0.324	0.1249	0.324	0.2471	0.000	0.2471	0.000	0.2471	0.000
Traffic	0.0154	0.878	0.0154	0.878	0.0154	0.878	0.0154	0.878	0.0154	0.878	0.0154	0.878
Mould/dampness	0.0085	0.942	0.0085	0.942	0.0085	0.942	0.0085	0.942	0.0085	0.942	0.0085	0.942
Pets	-0.0172	0.855	-0.0172	0.855	-0.0172	0.855	-0.0172	0.855	-0.0172	0.855	-0.0172	0.855
Maternal smoking during pregnancy	0.4697	0.000	0.4697	0.000	0.4697	0.000	0.4697	0.000	0.4697	0.000	0.4697	0.000
Paternal smoking during pregnancy	0.4854	0.000	0.4854	0.000	0.4854	0.000	0.4854	0.000	0.4854	0.000	0.4854	0.000
Early ETS	0.5897	0.000	0.5897	0.000	0.5897	0.000	0.5897	0.000	0.5897	0.000	0.5897	0.000
Current smoker(s)	0.6998	0.000	0.6998	0.000	0.6998	0.000	0.6998	0.000	0.6998	0.000	0.6998	0.000
Indirect effects of PE on outcome through:												
Obesity	0.0316	0.456	0.0165	0.807	0.0144	0.599	0.0317	0.456	0.0174	0.800	0.0153	0.580

Crowding index	0.0337	0.882	-0.2397	0.549	0.1221	0.357	-0.0224	0.920	-0.3356	0.426	0.0836	0.562
Number of children	0.0650	0.546	0.0916	0.556	-0.0142	0.598	0.0481	0.057	0.0663	0.050	0.0074	0.552
Traffic	0.0012	0.964	-0.0006	0.988	0.0012	0.943	0.0014	0.959	-0.0004	0.992	0.0012	0.942
Mould/dampness	0.0006	0.974	0.0001	1.000	0.0034	0.937	0.0006	0.976	0.0001	0.999	0.0034	0.937
Pets	0.0049	0.835	-0.0013	0.957	0.0042	0.831	0.0053	0.831	-0.0008	0.971	0.0042	0.831
Maternal smoking during pregnancy	0.2407	0.021	0.3431	0.093	0.0218	0.766	0.2347	0.023	0.3379	0.097	0.0238	0.747
Paternal smoking during pregnancy	-0.0602	0.540	-0.0195	0.912	-0.0269	0.575	-0.0498	0.613	-0.0052	0.977	-0.0263	0.585
Early ETS	0.2253	0.070	0.2079	0.443	0.1981	0.029	0.2141	0.087	0.1949	0.472	0.1973	0.027
Current smoker(s)	-0.0387	0.760	-0.3271	0.266	-0.1151	0.211	-0.0464	0.717	-0.3448	0.246	-0.1179	0.203

Table S5. Direct and indirect effects on physician diagnosed asthma (PDA) using different cut-off for defining parental education. ETS: Environmental Tobacco Smoke.

	**no education and primary school vs lower secondary school or above**		**no education and up to high school vs university degree and doctorate**	
	Coefficient	*P-value*	*Coefficient*	*P-value*
Direct effects on physician diagnosed asthma (PDA):				
Parental education (PE)	0.0061	0.982	-0.3676	0.127
Obesity	0.2551	0.205	0.2623	0.193
Crowding index	0.0156	0.930	0.0520	0.771
Number of children	0.2938	0.094	0.2910	0.096
Traffic	0.0920	0.633	0.0855	0.657
Mould/dampness	0.0604	0.795	0.0616	0.791
Pets	-0.2937	0.137	-0.2971	0.133
Maternal smoking during pregnancy	0.5003	0.034	0.5135	0.029
Paternal smoking during pregnancy	-0.1039	0.584	-0.0994	0.601
Early ETS	0.3632	0.075	0.3686	0.071
Current smoker(s)	-0.0623	0.754	-0.0504	0.800
Direct effects of PE on:				
Obesity	0.2411	0.199	0.2400	0.208
Crowding index	1.3755	0.000	1.3197	0.000
Number of children	1.1636	0.000	0.2908	0.052
Traffic	0.0056	0.975	-0.2511	0.103
Mould/dampness	0.1308	0.521	0.1321	0.505
Pets	-0.2036	0.244	-0.0770	0.610
Maternal smoking during pregnancy	0.5983	0.003	0.8379	0.004
Paternal smoking during pregnancy	-0.0434	0.764	0.5062	0.000
Early ETS	0.3881	0.010	0.7175	0.000
Current smoker(s)	0.8014	0.000	0.6558	0.000
Indirect effects of PE on PDA through:				
Obesity	0.0615	0.405	0.0629	0.448
Crowding index	0.0215	0.931	0.0686	0.782
Number of children	0.3419	0.165	0.0846	0.257
Traffic	0.0005	0.990	-0.0215	0.745
Mould/dampness	0.0079	0.879	0.0081	0.852
Pets	0.0598	0.432	0.0229	0.722
Maternal smoking during pregnancy	0.2993	0.059	0.4303	0.049
Paternal smoking during pregnancy	0.0045	0.893	-0.0503	0.614
Early ETS	0.1409	0.133	0.2645	0.081
Current smoker(s)	-0.0499	0.743	-0.0331	0.806

Table S6. Direct and indirect effects on current asthma (CA) using different cut-off for defining parental education. ETS: Environmental Tobacco Smoke.

	**no education and primary school vs lower secondary school or above**		**no education and up to high school vs university degree and doctorate**	
	Coefficient	*P-value*	*Coefficient*	*P-value*
Direct effects on current asthma (CA):				
Parental education (PE)	-0.4762	0.443	-0.4144	0.335
Obesity	0.1380	0.725	0.1340	0.733
Crowding index	-0.3207	0.363	-0.3042	0.392
Number of children	0.4452	0.182	0.4193	0.207
Traffic	-0.0259	0.946	-0.0349	0.927
Mould/dampness	0.0135	0.976	0.0165	0.971
Pets	0.0492	0.888	0.0506	0.884
Maternal smoking during pregnancy	0.7267	0.097	0.7282	0.096
Paternal smoking during pregnancy	-0.0372	0.918	-0.0142	0.969
Early ETS	0.3165	0.426	0.3205	0.420
Current smoker(s)	-0.4974	0.202	-0.5039	0.196
Direct effects of PE on:				
Obesity	0.2411	0.199	0.2400	0.208
Crowding index	1.3755	0.000	1.3197	0.000
Number of children	1.1636	0.000	0.2908	0.052
Traffic	0.0056	0.975	-0.2511	0.103
Mould/dampness	0.1308	0.521	0.1321	0.505
Pets	-0.2036	0.244	-0.0770	0.610
Maternal smoking during pregnancy	0.5983	0.003	0.8379	0.004
Paternal smoking during pregnancy	-0.0434	0.764	0.5062	0.000
Early ETS	0.3881	0.010	0.7175	0.000
Current smoker(s)	0.8014	0.000	0.6558	0.000
Indirect effects of PE on CA through:				
Obesity	0.0333	0.782	0.0322	0.805
Crowding index	-0.4411	0.403	-0.4015	0.422
Number of children	0.5181	0.191	0.1219	0.330
Traffic	-0.0001	0.999	0.0088	0.949
Mould/dampness	0.0018	0.997	0.0022	0.987
Pets	-0.0100	0.931	-0.0039	0.963
Maternal smoking during pregnancy	0.4348	0.132	0.6102	0.142
Paternal smoking during pregnancy	0.0016	0.979	-0.0072	0.967
Early ETS	0.1228	0.506	0.2299	0.446
Current smoker(s)	-0.3986	0.283	-0.3305	0.259

Table S7. Direct and indirect effects on current allergic rhinitis (CAR) using different cut-off for defining parental education. ETS: Environmental Tobacco Smoke.

	**no education and primary school vs lower secondary school or above**		**no education and up to high school vs university degree and doctorate**	
	Coefficient	*P-value*	*Coefficient*	*P-value*
Direct effects on current rhinitis (CAR):				
Parental education (PE)	0.2288	0.230	0.0439	0.809
Obesity	0.1145	0.437	0.1202	0.414
Crowding index	0.0887	0.477	0.1008	0.420
Number of children	-0.0794	0.530	-0.0640	0.611
Traffic	0.0765	0.573	0.0758	0.576
Mould/dampness	0.4009	0.009	0.4028	0.008
Pets	-0.2357	0.079	-0.2399	0.073
Maternal smoking during pregnancy	0.0441	0.814	0.0494	0.792
Paternal smoking during pregnancy	-0.0516	0.694	-0.0608	0.643
Early ETS	0.3388	0.020	0.3376	0.021
Current smoker(s)	-0.1754	0.206	-0.1660	0.230
Direct effects of PE on:				
Obesity	0.2411	0.199	0.2400	0.208
Crowding index	1.3755	0.000	1.3197	0.000
Number of children	1.1636	0.000	0.2908	0.052
Traffic	0.0056	0.975	-0.2511	0.103
Mould/dampness	0.1308	0.521	0.1321	0.505
Pets	-0.2036	0.244	-0.0770	0.610
Maternal smoking during pregnancy	0.5983	0.003	0.8379	0.004
Paternal smoking during pregnancy	-0.0434	0.764	0.5062	0.000
Early ETS	0.3881	0.010	0.7175	0.000
Current smoker(s)	0.8014	0.000	0.6558	0.000
Indirect effects of PE on CAR through:				
Obesity	0.0276	0.584	0.0289	0.638
Crowding index	0.1220	0.488	0.1330	0.438
Number of children	-0.0924	0.506	-0.0186	0.620
Traffic	0.0004	0.989	-0.0190	0.767
Mould/dampness	0.0524	0.608	0.0532	0.518
Pets	0.0480	0.370	0.0185	0.617
Maternal smoking during pregnancy	0.0264	0.805	0.0414	0.760
Paternal smoking during pregnancy	0.0022	0.899	-0.0308	0.549
Early ETS	0.1315	0.060	0.2422	0.015
Current smoker(s)	-0.1406	0.174	-0.1089	0.261
